# Lived experiences of caregivers with a family member living with a severe mental health condition in South Africa

**DOI:** 10.1186/s12888-025-06989-9

**Published:** 2025-07-16

**Authors:** Bongwekazi Rapiya, Nelisiwe Khuzwayo, Laura Asher, Carrie Brooke-Sumner

**Affiliations:** 1 Discipline of Public Health Medicine, School of Nursing and Public Health, College of Health Sciences, University of KwaZulu-Natal, Durban, South Africa; 2 Mental Health, Alcohol, Substance Use & Tobacco Research Unit, South African Medical Research Council, Francie Van Zijl Drive, Parow Valley, Cape Town, 7501 South Africa; 3 Nottingham Centre for Public Health and Epidemiology, School of Medicine, University of Nottingham, Nottingham, UK; 4 Institute of Mental Health, University of Nottingham, Nottingham, UK

**Keywords:** Severe mental health condition, South Africa, Family member, Caregiver, Schizophrenia, Lived experiences

## Abstract

**Background:**

In low- and middle-income countries (LMIC) such as South Africa, people with severe mental health conditions (SMHC) typically reside with family members, who serve as their primary caregivers. This study aimed to explore the lived experiences, needs and coping strategies of caregivers with a family member with SMHC in a low-resource setting in South Africa, and their perspectives on the provision of a support group programme.

**Methods:**

An exploratory qualitative study was conducted, which included 22 in-depth individual interviews, 15 of them being females and 7 males ranging from 26 to 72 years of age. These caregivers were recruited through the service user presenting at the health facility. The interview guide covered caregiving experiences, coping strategies, perceptions on recovery, and acceptability of peer-led mutual support groups. Written informed consent was obtained before conducting interviews. Interviews were audio recorded, translated from isiXhosa to English and transcribed. Thematic analysis using NVivo 12 was used to analyze findings.

**Results:**

Caregivers reported being socially excluded from family and community gatherings, as well as experiencing financial and emotional hardship because of their responsibilities. This influenced their overall well-being and ability to cope. Inadequate time for their own social activities and fulfilment were frequently described, and this was linked to their level of responsibility for the needs of others in their homes, as caregiving obligations were rarely shared among family members. Although some caregivers had developed ways to cope, such as hobbies and spiritual endeavours, some nevertheless reported less healthy coping strategies, including alcohol use. Whilst most caregivers indicated they would be interested in a peer-led mutual support programme, some said they would not be able to participate because of work or other responsibilities.

**Conclusion:**

Caregivers of people with SMHC in South Africa face considerable challenges, and supportive interventions are needed. Peer-led mutual support groups may hold the potential for providing this support.

**Supplementary Information:**

The online version contains supplementary material available at 10.1186/s12888-025-06989-9.

## Background

In South Africa, as elsewhere, severe mental health conditions (SMHC), such as schizophrenia, not only contribute to the overall burden of disease but are also associated with violations of human rights for people with the conditions and their family members. In addition, premature death associated with these conditions makes it a public health priority [1]. People with SMHC may benefit from antipsychotic medication [2, 3] (which was available in the district of the current study). Although there is access to free antipsychotic medication in some parts of South Africa, medication supply can be unreliable. However, people with SMHC also require other support that is not biomedical. Personal recovery moves beyond reduction in symptoms (clinical recovery) and is the process whereby the person realigns themselves to discover a hopeful and productive way of life despite the possible limitations of the condition. With the proper assistance, people with SMHC can make important progress towards recovery in a clinical and personal sense, but without support recovery can be hampered. Poverty is a known risk factor for the worsening of mental health conditions [4–7] and many people with SHMC and their families in South Africa may experience financial stress [8–10] and poor living conditions [11]. Many people in South Africa (including this study’s participants) reside in low-income metropolitan areas, which are marked by crowded living conditions, inadequate housing and sanitation, high rates of crime and violence, unemployment, poverty, and food insecurity, all of which may compound mental health conditions.

Deinstitutionalization (relocating patients with mental illnesses out of inpatient psychiatric hospitals and into the community) has caused a shift in the configuration of caring roles in South Africa as elsewhere. Whereas in the past most care was provided by healthcare professionals, particularly nurses, in institutional settings, the move to a more integrated, community-based approach means family members now act as primary caregivers [12]. Families are then by necessity a crucial source of care and support for recovery in low-income areas with few formalized government social security resources [13]. Allocation of resources for community mental health for SMHC in South Africa is constrained, with the majority being dedicated to inpatient care [14], and service users with SMHC are often repeatedly admitted and discharged at great cost to the public health system. To avoid this ‘revolving door phenomenon’, there have been calls for redirecting resources previously allocated to hospitals into community-based supportive services [15, 16], which would include support for families, but progress in this regard has been slow.

Being a family caregiver of a person with SMHC can be a rewarding experience that brings meaning and purpose to the caregiver’s life [17]. It can also be demanding, with the potential for physical strain, emotional strain, financial strain, loss of personal time and freedom, and risk of burnout [18, 19]. Caregiver burden is “the extent to which caregivers perceive the adverse effect that caregiving has on their emotional, social, financial, and physical functioning” [20]. This includes the tasks required to care for the service user and the extent to which the caregiver “minds” performing these tasks [20]. There may be tangible costs arising from mental health conditions, such as the collapse of a structured family life and loss of finances due to healthcare expenditure. In addition to these potential stressors, caregivers may also experience associated mental health stigma (from being linked to a person with SMHC), negatively impacting quality of life [21].

Caregivers of people with SMHC are an important and under-researched group in LMIC and South Africa. The current study explored the lived experiences of caring for a person with SMHC in a low-resource South African setting. This study was nested in the PRIZE study (Peer-led recovery groups for people with psychosis in South Africa) [22]. The overall aim of PRIZE was to provide support for people with SMHC and those who care for them through peer-led recovery groups [23, 24]. The current study describes formative research that was conducted with the aim of informing the development of these recovery groups so that they would be responsive to the experiences and needs of caregivers.

### The theoretical framework underpinning the study

Bronfenbrenner’s Theory of Ecological Systems underpinning this study offers a comprehensive lens to understand the lived experiences of caregivers with a family member living with a SMHC in South Africa. This theory emphasizes the intricate interplay between individuals and their environments, illustrating how multiple systemic levels influence one’s development and daily experiences.

In the context of caregivers, the **microsystem** refers to the most immediate environment and encompasses interactions with family members, friends, healthcare professionals, and support networks [25]. These close relationships significantly impact the caregiver’s emotional well-being and coping strategies. The **mesosystem** captures the connections between various microsystems, such as interactions between healthcare providers and family dynamics [25]. Effective communication and coordination among these stakeholders can alleviate caregiver burden, while a lack of support can exacerbate stress. The **exosystem** reflects broader social influences indirectly affecting caregivers, such as workplace policies, societal attitudes towards mental health, and community resources. Limited access to mental health services, inadequate financial support, and stigma can intensify caregivers’ challenges [26]. The **macrosystem** includes cultural norms, societal values, and government policies [27]. In South Africa, structural inequalities, economic disparities, and the limited integration of mental health in public health policy play a pivotal role in shaping caregivers’ experiences. Finally, the **chronosystem** considers how historical and temporal contexts, such as past traumas or ongoing socioeconomic struggles, affect caregiving experiences [28]. These layers illustrate that caregiving for a family member with SMHC is not merely a personal experience but is deeply embedded within broader social, cultural, and economic contexts.

## Methods

### Setting

The study was conducted in low-resource urban and semi-urban areas of Nelson Mandela Bay Metropolitan Municipality, Eastern Cape Province, South Africa. The Biomedical Research Ethics Committee (BREC) of the University of KwaZulu-Natal (BE407/13; HSS/0623/012D) and the South African Medical Research Council Ethics committee (EC007-4/2019) gave ethical approval for the study. Participants were recruited from six primary health care facilities which provide services to mental health care users in this district. These clinics were the point of contact with service users with SMHC, through which primary caregivers were contacted and recruited for the study. In this study, the primary caregiver was an individual or family member who is primarily active in caring for the service user. After consultation with service users, they would identify the person whom they regarded as their primary caregiver. Before data collection, service users from these clinics were given information sheets to share with the person whom they considered their primary caregiver, after which written informed consent was gained from the caregiver during a home visit at which participation in the study was discussed.

### Data collection

Only those who met the criteria of primary caregivers of service users living with SMHC that have not been hospitalised, for their mental disorder in the past 3 months and was not experiencing active symptoms of their disorder, be years and older, isiXhosa speaking (target population for PRIZE recovery groups and the main cultural group in the study district) and willing to give voluntary informed consent were included in the study. Before data collection, service users from these clinics were given information sheets to share with the person whom they considered as their primary caregiver, after which voluntary written informed consent was gained from the caregiver. Consent was sought after a clear explanation of the study in a comprehensible language, explaining the risks and benefits of the study, confidentiality of information collected, and that participation is voluntary. In-depth interviews were conducted in isiXhosa with each participant by the main researcher (BR) and Research Assistant (HM), who were both female first language isiXhosa speakers and experienced qualitative interviewers. Interviews lasted an hour on average and were audio recorded with the participants’ consent. They were conducted in a private space in the participant’s home or at a local venue. An interview guide (Supplementary File 1) was used which included questions such as: How does caring for your relative with a mental health condition affect your life? How do you cope with your family member’s health condition in your day-to-day life? The assignment of identifying codes protected the confidentiality of respondents’ data throughout the analysis and write up processes.

### Analysis

Interviews were transcribed and translated into English by BR and a Research Assistant (HM), contributing to the initial familiarization process. Interviews were stored on password-protected computers and NVivo 12 was used to manage the data. A thematic data analysis was conducted using Braun and Clarke’s method [29] in which a coding framework was first developed iteratively, and then applied to the whole dataset. Codes were then grouped into themes and an overall thematic framework created through discussion between BR and CBS. This study was developed and is presented in line with COREQ guidance for reporting qualitative research [30].

### Issues of trustworthiness

Lincoln and Guba [31] define a study’s “trustworthiness” as the internal verification, external verification, reliability, and objectivity. They rely on four general criteria in their approach of trustworthiness [32]. These are credibility, transferability, dependability, and confirmability [32]. Trustworthiness is essential in qualitative research to establish these four general criteria (credibility, transferability, dependability, confirmability), and overall rigor of the study [33]. Ensuring the trustworthiness of the data enhances the reliability and validity of the study [34]. This study applied these criteria. **Credibility** was done by member checking, also referred to as participant or respondent verification. This was done by the main researcher with 5 caregivers where they were given an opportunity to give feedback on themes generated from the data they provided from the interviews [35]. T**ransferability** was attained by examining and discussing the results of this study in relation to caregiving in broader literature, and findings may apply to studies that will be undertaken in the future on a related subject. Dependability was achieved by checking the translations and transcriptions of the isiXhosa recordings which were done by an independent translation agency to see if the translation process jeopardized the data authenticity and dependability. There were no differences in the meanings produced by the translated transcripts. **Confirmability** was promoted by utilizing a second coder to assure confirmability. The researcher used r**eflexivity** to prevent her personal opinions, judgments, and behaviours from distorting the research, the researcher constantly reviewed them throughout the research process. Also, she looked at how the research may have influenced her and possibly changed her outlook on the research topic. Consent procedures were modified and simplified to ensure participants understood the study information, as detailed in the informed consent section. The lead researcher recognized her own biases as she was conducting the data analysis [36]. Lastly **positionality** required the researcher to evaluate their own position in respect to three factors: the issue under examination, the participants in the study, and the research methodology, context, and procedure [37, 38]. In this case, the researcher’s position was in-between. By employing linguistic meanings that the researcher understood, caregivers were able to express themselves completely and freely without having to go in-depth to explain things. Although the researcher did not belong to the same socioeconomic category as the participants, the researcher was able to empathize with the caregivers’ current circumstances since she had previously held a similar position. This allowed the caregivers to communicate their emotions, desires, and hopes without holding anything back. The researcher had the benefit of being able to note if participants were feeling uneasy throughout the interviews and would refer for further support as necessary because of the years of expertise conducting interviews for research and interacting with vulnerable participants. Years of experience conducting qualitative research enabled the researcher to see how crucial it is to encourage caregivers to express themselves honestly without making them feel as though their opinions are unimportant, which facilitated the collection of rich data. While perceived as an outsider, the researcher was more accessible because of her own positionality as a mother and having cared for someone with a SMHC. The researcher made it clear during the interviews that she is not a service provider (i.e., a psychologist or social worker).

## Results

### Sociodemographics of participants

Interviews were conducted with a total of 22 primary caregivers of service users living with SMHC. Of the 22 participants, fifteen were females and seven were males and their ages ranged from 26 to 72 years (Refer to Table 1). Most were taking care of a close relative (service user) which was either a sibling or their child and only one caregiver was taking care of a parent. Most of the caregivers have been primary caregivers since the service user’s first diagnosis and many were also taking care of other family members (e.g. grandchildren or a sick parent or sibling) other than the service user. Only one caregiver was taking care of two siblings who were both diagnosed with SMHC. Many of these caregivers were not working and were solely relying on the monthly disability grant (US$109) that is available to people with enduring psychosocial disabilities from the South African Social Security Agency, or the monthly old age grant (US$109). All had some level of schooling with 20% having some primary education, 68% some secondary education and 12% having some tertiary education.

**Table 1:** Sociodemographics of study participants

	N (total n = 22)	%
**Sex**		
Female	15	68%
Male	7	32%
**Age range**		
26–35 years	4	14%
36-50 years	8	38%
51 years and older	10	48%

### Theme 1: experiences of caregiving

#### Subtheme 1: overall impressions of being a caregiver

When asked how caring for a family member with a mental health condition affects their lives, caregivers had positive and negative impressions indicating variability in experiences. As the only caregiver for their family members, some caregivers said it had not been an easy path but one into which they had grown and adjusted. For a variety of reasons, including taking care of other family members, caregiving was burdensome for some caregivers as they were not getting help from the immediate family and providing care tended to have more negative than positive moments. Among the negatives were the stigmatization and discrimination of the service users by community members.

*“Yes*,* sometimes they fear him*,* and they would call the police on him because he is in and out. This is his home and there it is his aunt’s house. So*,* he is in between these homes because he is scared”. (Caregiver PID 22*,* female [*57*]*)

Others experienced positive outcomes in relation to caregiving. These included the service user helping with house chores, doing things independently and the service user seeking work to supplement the grant they were receiving. This could have been because of their hands-on approach and the support they were giving to their family members.

*“When she must go to the clinic*,* she is quite good. We don’t monitor her. She goes by* *herself on her dates. Most of the time she does not even ask for a bus fare because she walks*,* which is also good. (Caregiver PID 35*,* female *[41])

*“So*,* she is a person that buys food*,* she buys groceries*,* the fridge must have meat. (Caregiver PID 23*,* female *[47])

#### Subtheme 2: practical aspects of caregiving

Some caregivers noted it was part of their responsibility to assist and support family members with practical aspects of life including cooking, cleaning, and maintaining personal hygiene. Some noted that their family member needed reminding of aspects of personal care and to take medication. Others noted that their family member was more independent and was able to contribute to the practical activities of the household, though some emphasized that getting their family members to do these daily activities required some convincing and negotiating.

*“I will have to tell him what he must do. At times he would ask “what are we cooking today?” I would normally cook and at times he would cook. I would say “I am tired”. He will say “what should I cook? I would then tell him what he must cook. He would cook that. If I give instructions and leave*,* I will come back*,* and that thing is done.” (Caregiver PID 58*,* male *[32])

*“It is not easy*,* but it’s easy with this one because he is not aggressive. When I tell him to take his medication*,* he takes it. I make sure he takes his medication… He used to get lost*,* and I would not know where he was sleeping. Ever since he came to the clinic*,* they took his blood and he was given medication*,* and he is alright now. He doesn’t trouble me with anything”. (Caregiver PID51*,* female *[71])

#### Subtheme 3: understanding services users’ needs for care

Some participants described that over time they had developed awareness of when their family member needed further support (even if they did not express this verbally). Years of experience caring for a family member with SMHC had made most of the caregivers aware of the specific early warning signs that suggested their family member might have been becoming more unwell. These caregivers were prepared to address this need for care before it worsened since they had experienced these circumstances several times. They noted that during these times the care, attention and presence their family member required were amplified, and the responsibility and burden they felt increased. On the other hand, some caregivers described relying on their family member to tell them when they needed additional care.

*“He opens the lock at 6am*,* I would ask “What woke you up?” He would say*,* “I woke up around 3 am*,* I bathed myself”. He cleans the yard*,* picks up papers*,* that’s how I see there is something wrong*,* because he is a not a very active person”. (Caregiver PID 27*,* female *[45])

#### Subtheme 4: caregivers support accessing healthcare and other services

While some caregivers described their perception that they were able to prevent situations which would lead to their family member’s relapse (e.g. by avoiding stress in the household), for some, this was not possible when their family member became more unwell and was combative and inflexible. To ensure that the required assistance was obtained, this frequently necessitated the caregivers requesting an ambulance or the South African Police Services (SAPS) to accompany the family member to hospital. Several caregivers did report that once they were able to get to the hospital, they would get help quickly.

*“If he gets sick*,* we must call the ambulance or the police. The police will take him to [name] hospital. They [hospital staff] do assist us… they do”. (Caregiver PID 22*,* female *[57])

#### Subtheme 5: recovery as a route to reduced caregiver burden

For most caregivers, their hope and ideas on what recovery would look like for their family member centred around their being able to get a job or have an income and be independent or be a contributing member of the household financially. Some caregivers mentioned that this would also mean that they as caregivers would have the chance to accomplish activities that they had previously been unable to do because they were responsible for caring for their family member.

*“I would be relaxed*,* no depression and no stress and be normal*,* there is nothing else. I want him to work*,* because I also want him to have a wife and children and all of that*,* the normal life I know. That is the progress that I want in his life” (Caregiver PID 34*,* male *[47])

### Theme 2: challenges associated with caregiving

#### Subtheme 1: lack of involvement of other family members

An important source of support for caregivers might have been the involvement of other family members in the care of a person with SMHC. Most participants however described fulfilling their caregiving role alone, with little to no assistance from other family members. One caregiver felt that this is something that could be shared amongst family members but in her case, she was the only one doing it.

“*My mother gave birth to three children; I am the only child who is looking after her…I feel like I am not getting any considerable support from them [siblings]. (Caregiver PID 18*,* female *[34])

#### Subtheme 2: social isolation

Several caregivers spoke of how not only their family members with SMHC, but they themselves were isolated and had fewer social contacts than before they had the caregiving role. Caregivers also described the stigma due to their family member’s condition, adding that this discouraged them from attending functions, traditional ceremonies, and other events in their community because of fear of how they may be treated.

*“No one will want to come help you*,* it becomes your own burden to you as a caregiver. Otherwise*,* anyone… they don’t help…. they don’t help*,* even those people from church*,* those maybe who can come visit and give support and show love”. (Caregiver PID 10*,* female *[63])

#### Subtheme 3: financial responsibilities as a primary source of stress

Financial responsibilities were a significant source of stress for most caregivers. For some, caregiving had led to loss of income, increased expenses for medical supplies and transportation, and other financial strains. Caregivers reported financial stress as their most important stressor and described the toll financial problems had on their wellbeing. Some caregivers revealed that they care for other family members in addition to their family member with SMHC. These additional family members were grandchildren and the elderly who required assistance in meeting their basic needs. The result was caregivers sometimes had financial responsibility for entire households, with providing food for multiple family members becoming a key concern. Elderly caregivers who received a national pension also reported having responsibility for financially supporting their adult children who were unemployed.

*“My stress is caused by me being unemployed when I have children and grandchildren. My children are not working as well. My worry is that if anything could happen and I do not even have a funeral cover*,* you know”? (Caregiver PID 22*,* female *[57])

#### Subtheme 4: inadequate time for social activities, relaxation, and fulfilment

The range of demands and responsibilities caregivers were managing reportedly left little time for their own social activities, and opportunities for relaxation and fulfilment. This was particularly challenging for caregivers who provided full-time care and had limited access to resources or support. Caregivers described difficulties in finding ways to take care of themselves and prioritize their needs to maintain their well-being. Some caregivers missed out on participating in daily activities in their communities or with their families because they felt the responsibility to protect their family member with SMHC. They described neglecting their own needs while prioritizing those of their family member. This focus on the well-being of their family member meant performing certain duties even if they were physically or emotionally exhausted, which they undertook from a sense of duty as family leaders to take care of, provide for, and protect their families.

*“The struggles that we go through while we are trying to make these people we are taking care of happy. We sacrifice things. I am [name]’s parent. I used to ask*,* “when do I live? I could be doing things for myself” (crying). I must think that winter is coming up. I must lay bye [buy on credit] stuff for me and [name]. So*,* I sacrifice stuff for my siblings”. (Caregiver PID 58*,* male *[32].

### Theme 3: coping strategies

#### Subtheme 2: healthy and unhealthy coping strategies

Caregivers described a range of coping strategies including healthy and unhealthy coping strategies. Healthy strategies included seeking support from others, practicing self-care through exercise and involvement in religious activities, and communicating effectively with their loved ones and healthcare providers. Unhealthy strategies included using alcohol and over-the-counter medications and binge eating to cope with the stress of caregiving.

*“When you stress*,* you think about everything like your veins are throbbing in a sore way*,* so I would feel like taking pain killers and sleep. Yes*,* I would take pills or drink water when I don’t have money to buy pills and then sleep”. (Caregiver PID 29*,* female *[50])

*“I read my bible and sit outside if I want to sit outside. There is not much stress*,* because I always tell myself that I am who I am*,* and I am not going to be like anyone else”. (Caregiver PID 37*,* female *[65])

#### Theme 4: acceptability of peer-led support groups

Participants felt that social support is crucial for them and their family members to achieve mental health and wellbeing. They viewed mutual support groups as a way that caregivers could access social and practical support. Caregivers perceived these groups as an acceptable way of obtaining support for several reasons. Firstly, some felt that they would appreciate the opportunity to connect with others who are in similar situations and who understand the unique challenges of caregiving. Caregivers felt groups could be a place that can provide a sense of community and belonging, which they felt could be particularly important for them as they sometimes feel isolated or overwhelmed by their caregiving responsibilities. In addition to providing social support, these groups were seen as something that could offer practical support, such as information and resources related to caring for someone with SMHC. This included information about treatment options, financial assistance, and other resources that can help caregivers navigate the challenges of caregiving.

*“Sit in one place*,* have a group where we give each other advice. We could leave the people we are looking after or come with them to the groups*,* cook*,* have fun and get that support”. (Caregiver PID 22*,* female *[57])

## Discussion

This study used qualitative methods to explore the lived experience of caregivers supporting people with a SMHC and living in a low-resource setting of the Eastern Cape Province of South Africa. The study aimed to contribute to the research gap which is the the lack of research regarding support for caregivers of this vulnerable group, and to the tailoring of a peer-led recovery support programme for people with SMHC and caregivers (PRIZE) [24]. PRIZE will contribute evidence for community-based psychosocial support for people with SHMC and their families in an area where these services are not currently available [23, 24].

The current study sheds light on the experiences of caregivers of individuals with SMHC and the subsequent impact on their overall wellbeing. This adds to literature that describes the role of family caregivers in LMIC and the impacts they experience due to the caregiving role [39–42]. As in other LMIC [43, 44], caregivers in the current study play a crucial role in supporting their family members through assisting them in practical aspects of daily living and meeting their basic needs. Findings from the current study align with those from other LMIC showing the responsibility of caregivers becomes especially demanding when their relatives become more unwell, requiring them to be fully present and attentive to their family member’s needs [45–47]. Perhaps the most notable finding from this study is the burden and stress experienced by caregivers due to the nature of their responsibilities. Participants described the sense of overall responsibility for their family members’ well-being and findings highlight the substantial impact of financial responsibilities as is the case in other LMIC in which community-based support services are inadequate [48–50]. The financial aspect of caregiving has emerged as a key source of stress for caregivers in other settings, particularly when they are responsible for covering the costs associated with multiple responsibilities (e.g., other family members) [51–53]. Worries about financial problems further compound the already demanding nature of caregiving responsibilities [47, 48] leading to a sense of being overwhelmed. Social transfers in the form of grants and pensions provide some measure of decreasing poverty [54] but participants in this study highlighted the inadequacy of this support.

With this backdrop, caregivers commonly described neglecting their own self-care and personal needs while prioritizing the care of their family member through a sense of duty, a pattern seen in other settings where family members are the main source of support [40, 48, 55]. Psychological well-being of caregivers can be strongly influenced by this lack of self-care [56, 57] and in the long term can result in serious consequences for caregivers’ overall health and well-being, impacting their ability to provide effective support [56, 57]. Participants also described impacts on their social wellbeing in that their responsibilities limited their ability to engage in social activities and maintain relationships, a challenge identified by caregivers in other LMIC settings [58, 59]. Some expressed emotional anguish because of an absence of support from their relatives and continuing tension around family circumstances, as reported in other settings [46, 56, 60].

In view of these experiences, participants described a variety of coping strategies to manage their challenges. The role of supporting someone with an SMHC may lead to chronic stress [53] and either healthy or unhealthy coping strategies. While some caregivers adopted healthy coping strategies (e.g. support from religion), others resorted to unhealthy behaviours that can be potentially harmful (substance use), a situation that is seen also in other LMIC countries [61, 62]. Caregivers can be supported to handle stress more efficiently and enhance their overall well-being through fostering appropriate coping techniques [63, 64]. Findings from this study also highlighted that some caregivers experience positive outcomes related to caregiving. The caregiving role can strengthen family bonds and give a space for development of meaningful family relationships [65, 66].

Caregivers spoke about peer support groups as a being welcome support, and they saw mutual support groups as a place they could share caregiving experiences and challenges and be able to connect with people like themselves. This aligns with literature showing that caregiver support groups can offer a safe environment for caregivers to discuss their personal situations, seek advice and develop healthier coping mechanisms [67, 68]. Peer support programs may also be helpful in improving hope, and self-confidence [69] in relation to the caregiving role.

Several recommendations stem from this study for the PRIZE programme and for wider provision of support for caregivers. Firstly, this study has shown that caregivers of people living with a SMHC face multiple challenges, therefore relevant stakeholders including non-governmental organisations in the community and government departments should collaborate to provide adequate support for these needs [70, 71]. This support should include assistance in managing problems effectively (including financial stressors) and promoting overall well-being through enhanced coping [72]. Peer support groups may be an appropriate avenue for delivering this support and may be beneficial for recovery for both the caregiver and family members [73]. Secondly, addressing the widespread financial challenges faced by caregivers is crucial to ensuring their well-being [74, 75] and enhancing the quality of care [76] provided to individuals with a mental health condition. Policy initiatives and further financial support mechanisms should be established to assist caregivers in managing their responsibilities. Feasible community-based economic empowerment initiatives for caregivers and families [74] are crucial in the South African context.

### Limitations

While it incorporated the principles of rigorous qualitative research, this study has several limitations. The inclusion of only caregivers of individuals with SMHC in some townships in the Nelson Mandela Metropolitan Municipality was a limitation of this study and this could mean that their shared experiences do not necessarily characterize other parts of South Africa with a similar setting. There could be a bias in that caregivers who were happier with the role of caregiving were more likely to agree to be interviewed and share their experiences than those who were not. Also, there could be a bias that the caregivers who were not happy with the role of caregiving, were most likely to volunteer to be interviewed with the hope of possible release from frustrations regarding caregiving and were also hoping to get help from being part of the study.

## Conclusion

From this study in the Eastern Cape region of South Africa, we have reported experiences and perspectives of family caregivers who are the primary caregiver for a person with a SMHC. The findings highlight the mental health, social, and financial difficulties faced by caregivers and the need for interventions to support caregivers as a key human resource in the provision of care for people with SMHC. Interventions to enhance social support and promote healthy coping may benefit entire family systems that play a crucial role in the lives of those with mental health conditions.

## Electronic supplementary material

Below is the link to the electronic supplementary material.

Supplementary Material 1

## Data Availability

The data from this study are available from the corresponding author on reasonable request. carrie.brooke-sumner@mrc.ac.za”.
