# Supplementary material for: Lived experiences of caregivers with a family member living with a severe mental health condition in South Africa
Source: BMC Psychiatry. 2025 Jul 16;25:706. doi: 10.1186/s12888-025-06989-9 (PMC12265331; doi:10.1186/s12888-025-06989-9)
Supplement: Supplementary file 1 — Supplementary Material 1 [file 12888_2025_6989_MOESM1_ESM.pdf]

SUPPLEMENT 1: Interview Schedule [English]

Supplement 1: In-Depth Interview Caregivers Interview Schedule

Lived experiences and needs of caregivers with a family member living with a severe mental health condition in South Africa.

INDIVIDUAL INTERVIEW GUIDE FOR CAREGIVERS OF MENTAL HEALTH SERVICE USERS

Note for interviewers:
Instructions for you are numbered and in BOLD.
Text for you to read to the participant is in normal text.

Date of interview	
Name of interviewer	
Participant ID	

1. **Introduction**

Thank you for taking time and agreeing to this interview. As you know, people living with a severe mental health condition in the community in South Africa need social support as well as medication prescribed by a psychiatrist to treat their symptoms. PRIZE is a research study with researchers from the South African Medical Research Council, working together with the Eastern Cape Department of Health. We are developing a programme of support that will involve social workers and also peer supporters (people with a severe mental health condition who can support others in their recovery journey). We want this programme to fit the needs of service users and their caregivers, so we want to hear from you about your experiences and what is important to you. This interview is about your experiences of caring for your family member, and how you would like things to improve. First, we would like to ask you some background information.

2.Sociodemographic questions (interviewer to tick as appropriate)

2.1 Note gender of patient, if not sure, ask:

How would you describe yourself?

Male	
Female	
Other	

2.2 What was your age at your last birthday __________years.

2.3 Note race of caregiver if not sure, ask.

How would you describe yourself?

African	
Coloured	
Asian/Indian	
White	
Other	
2.7 If other, specify	

2.4 What is your current marital status? (Indicate one only)

Married	
Living with partner	
Widow/widower	
Divorced or separated	
Never married (single)	
Other	
2.9. If other, specify	

2.5 What is the highest level of education you have passed?

Less than one year completed	
Sub A/Class 1/Grade 1	
Sub B/Class 2/Grade 2	
Standard 1/Grade 3	
Standard 2/Grade 4	
Standard 3/Grade 5	
Standard 4/Grade 6	
Standard 5/Grade 7	
Standard 6/Grade 8	
Standard 7/Grade 9	
Standard 8/Grade 10	
Standard 9/Grade 11	
Standard 10/Grade 12	
Further studies – incomplete	
Diploma/other post school studies –	
Degree	

Now we would like to ask a few questions about where you live and your work.

2.6 How would you describe your current living situation?

I have a place to live where I can stay as long as I want.	
I am living in a place, but may not be able to stay there in the future	
I do not have any regular place where I can live	
Other	

2.7 If responded “other” in 2.6., please specify: ___________________________

2.8 Which of the following best describes your current employment status?

Unemployed and looking for work	
Unemployed and not looking for work	
Employed part-time	
Employed full-time	
Self-employed	
Pensioner	
Student/Scholar/Learner	

2.9 What is your main source of income? (Where do you get most of your money from each month?)

Formal employment	
Self-employment	
Odd jobs	
Government grant (childhood/disability)	
Income from investments	
Maintenance (child support or money from ex-partner for living costs)	
Scholarship/student loan	
Pension	
No income	
Other	

2.10 If Other, specify.

2.11 On average, what is your monthly income.

Less than R600	
R600-1000	
R1001-2000	
R2001-4000	
More than R4000	
Don’t know	

Interview Section 1 Experiences of caregiving

Start a discussion with the participant using Question 1 below. Give them time to share their thoughts. Then use the probes to get them to talk more about the topic.

Question 1: How does caring for your relative with a mental health condition affect your life?

Probe: How were they first diagnosed and how did this affect you?

Probe: What things does your family member need help with?

a. Keeping clean and tidy?
b. Looking after the home?
c. Eating well?
d. Managing their money?
e. Using public transport?
f. Looking after their children?
g. Anything else?

Probe: How do you know if they need to go to hospital? What do you do when this happens?

Probe: Do they have any difficulties in accessing the health services that are available?

Probe: How does caring for your family member who has a mental health condition feel for you?

Probe: What other caregiving responsibilities do you have?

Probe: Are you working? What type of work do you do or what would you like to do?

Probe: What social activities do you miss out on because of caregiving?

Probe: Can you tell me about any times you have been treated badly by family, community members or health workers because of your family member's illness?

Interview Section 2 Coping strategies

Start a discussion with the participant using Question 2 below. Give them time to share their thoughts. Then use the probes to get them to talk more about the topic.

Question 2: How do you cope with your family member's illness in your day-to-day life?

Probe: Can you tell me about days that are better for you and what makes it better or worse?

Probe: Do you get any other help or support from other people in taking care of this person?

Probe: What causes you to feel stress in your daily life and how do you cope with this?

Probe: Can you tell me about any times you have been mistreated by the family member you care for?

Probe: Do you know of any services besides medication to help you and your family member?

Probe: Do you use alcohol, tobacco, or any other substances (e.g., dagga) to help you cope?

Probe: Does your use of substances affect you being able to keep up with your caregiving responsibilities?

Interview Section 3 Perceptions around recovery

Start a discussion with the participant using Question 3 below. Give them time to share their thoughts. Then use the probes to get them to talk more about the topic.

***Question 3: When we talk about recovery from a mental health condition we do not mean to be cured, but to live a fulfilling life. What would change in your life if the family member you care for was able to recover in the sense of functioning better at home and in the community*?**

Probe: What are the most important things that they would be able to do that would improve their and your lives?

Probe: What are the most important things that stop them from having good quality of life?

Probe: What are the most important things that stop YOU from having good quality of life?

Probe: How is your family member excluded from community activities or opportunities and how could this be changed?

Probe: Are there relationships with other people you feel you miss out on? What relationships would you like to build?

Interview Section 4 Acceptability of peer support groups

Start a discussion with the participant using Question 4 below. Give them time to share their thoughts. Then use the probes to get them to talk more about the topic.

Question 4: If you could imagine the best way to help caregivers like yourself, what would it be?

Probe: If there was a group where caregivers with family members with a mental health condition came together to share experiences, how would you feel about being part of that?

Probe: Would you prefer to be in a group with other caregivers only, or with caregivers and family members together?

Probe: What other commitments do you have that could make it difficult for you to attend a group?

Probe: What could be done to make sure caregivers, especially women, would be able to attend a group like this?

Probe: Where would you prefer groups to take place?

Probe: What would motivate you to keep going to the groups?

Probe: We could call someone who has the same experiences as you and also cares for someone with a mental health condition in a group a ‘peer supporter’. How would you feel getting information and support from a peer supporter, rather than, for example, a health worker?
Probe: What would you like to talk about in a group like this?

Probe: How would you feel about sharing personal experiences with the group? What would make you feel more comfortable sharing?

Probe: Which of your experiences would you like to share with others? How do you think this could help them?

Probe: How could a peer supporter be helpful for you? And for your family member?

Probe: If you could be involved in an income generating project, what type of work would you like to do?

Probe: In the PRIZE study, we plan to run groups, and, in the beginning, we would like to audio record the group discussions, so that we can listen to them, and help the group facilitator to improve. We will never share the recording with anyone outside of the research group. How would you feel about this if you were a group member?

Probe: Do you have anything to add that we have not covered in this interview?

Thank you very much for your time
